# Engineered induced-pluripotent stem cell derived monocyte extracellular vesicles alter inflammation in HIV humanized mice

**DOI:** 10.20517/evcna.2022.11

**Published:** 2022-04-24

**Authors:** Bing Sun, Scott Kitchen, Norina Tang, Andreas Garza, Sheela Jacob, Lynn Pulliam

**Affiliations:** ^1^San Francisco VA Health Care System, Department of Laboratory Medicine, San Francisco, CA 94121, USA.; ^2^UCLA AIDS Institute, Division of Hematology and Oncology, David Geffen School of Medicine, UCLA, Los Angeles, CA 94121, USA.; ^3^ATCC, Gaithersburg, MD 20877, USA.; ^4^University of California, San Francisco, Departments of Laboratory Medicine and Medicine, San Francisco, CA 94121, USA.

**Keywords:** Monocyte, extracellular vesicles, pluripotent stem cells, miRNAs, HIV, bone marrow-liver-thymic (BLT) mouse, inflammation, neuroinflammation

## Abstract

**Aim:**

A peripheral inflammatory response can drive neuroinflammation in a number of infections including human immunodeficiency virus (HIV). Monocyte/macrophage (M/Mφ) activation is a hallmark of acute HIV infection and a source of chronic inflammation in a subset of HIV-infected individuals. We sought to decrease peripheral inflammation and M/Mφ transmigration after HIV infection by engineering extracellular vesicles (EV) to antagonize a microRNA (miR) associated with inflammation. We hypothesized that induced pluripotent stem cell (iPSC)-derived monocyte EVs (mEVs), engineered to contain an antagomir to miR-155 (αmiR mEV) would target monocyte inflammation and influence neuroinflammation in an HIV-infected humanized mice.

**Methods:**

mEVs were characterized by tetraspanins, nanoparticle tracking analysis, electron microscopy, and their preferential entry into circulating monocytes as well as testing for endogenous selected miRNAs. HIV-infected humanized mice were treated with control or antagomir155 mEVs. Plasma viral load was measured plus activation markers on lymphocytes and monocytes and the number of macrophages in the brain was quantified.

**Results:**

mEVs preferentially entered peripheral monocytes. HIV infection increased C-C chemokine receptor type 5 (CCR5) and major histocompatibility complex, class II, DR (HLA-DR) expression on T cells and monocytes. Treatments with mEVs did not decrease plasma HIV viral load; however, mEVs alone resulted in a decrease in %CCR5+ and %HLA-DR+ on T cells and an increase in %CCR5+ monocytes. αmiR mEVs decreased %CCR5 on M/Mφ. The mEV-treated HIV-infected mice did not show an increase in macrophage transmigration to the brain.

**Conclusion:**

mEVs alone caused an unexpected decrease in lymphocyte activation and increase in monocyte %CCR5; however, this did not translate to an increase in macrophage transmigration to the brain.

## INTRODUCTION

Neurocognitive impairment associated with human immunodeficiency virus** (**HIV) infection is initially driven by peripheral inflammation. Activated monocytes produce cytokines and monocyte-derived macrophages (M/Mφ) transmigrate into the brain, seeding the brain with HIV and contributing to neuroinflammation. Quenching peripheral inflammation with antiretroviral therapy (ART) for most HIV-infected individuals decreases impairment except for a few that have lingering chronic inflammation that perpetuates neuroinflammation and potential cognitive impairment. While ART greatly contributes to lowering the viral load to undetectable and, in most individuals decreasing chronic inflammation, cognitive impairment continues for some.

Targeting chronic inflammation remains an active area of research for many diseases including HIV. New therapeutic agents that could suppress the immune system without immunosuppression, thereby exposing someone to increased susceptibility to infection, are needed. While targeting the virus with antiretrovirals is standard care, targeting inflammation as adjunct therapy may decrease susceptible cells as well as dampen the deleterious effects of inflammation. Targeting a specific pro-inflammatory cytokine has not had widespread acceptability (Review Ref.^[1,2]^), although an interferon inhibitor showed promise to restore immune function in humanized HIV-infected mice^[3]^. Newer approaches to modulating the immune system include using noncoding microRNAs (miRs). miRNAs transcriptionally control gene expression for many targets. Our previous studies identified miRs associated with monocytes activated by interferon alpha and lipopolysaccharide (LPS) (I/L) to mimic immune activation in HIV infection^[4,5]^. In those studies, miR-155 was elevated in I/L-treated monocytes and their extracellular vesicles (EVs). miR-155 is highly expressed in T and B cells and M/Mφ (Review Ref.^[6]^).

Based on these *in vitro* studies, we set out to determine if monocyte EVs could be engineered to reduce peripheral inflammation in the setting of HIV infection, lower viral load and decrease peripheral monocyte activation markers plus neuroinflammation as defined by M/Mφ infiltration to the brain. We used an HIV-infected humanized mouse model^[7,8]^. This involves the implantation of immunodeficient mice with human fetal thymus, liver and hematopoietic stem cells [termed the humanized bone marrow-liver-thymus (BLT)] mouse model^[8]^. This mouse has a functional human immune system that can be infected with HIV. We engineered induced pluripotent stem cell (iPSC)-derived monocyte EVs (mEVs) to overexpress antagomir-155 and used these engineered EVs to target monocytes in HIV-infected BLT mice. We found that these mEVs preferentially targeted peripheral monocytes in the mouse and that the antagomir-155 silenced miR-155; however, the mEVs did not decrease HIV viral load. mEVs alone suppressed C-C chemokine receptor type 5 (CCR5) expression on T cells and increased CCR5 on M/Mφ. In spite of an increase in CCR5 expression on monocytes, there was no increase in M/Mφ transmigration to the brain. Thus, we found that mEV treatment had distinct immunomodulatory effects *in vivo*.

### METHODS

### iPSC-monocyte culture and EV collection

**Figure 1:**
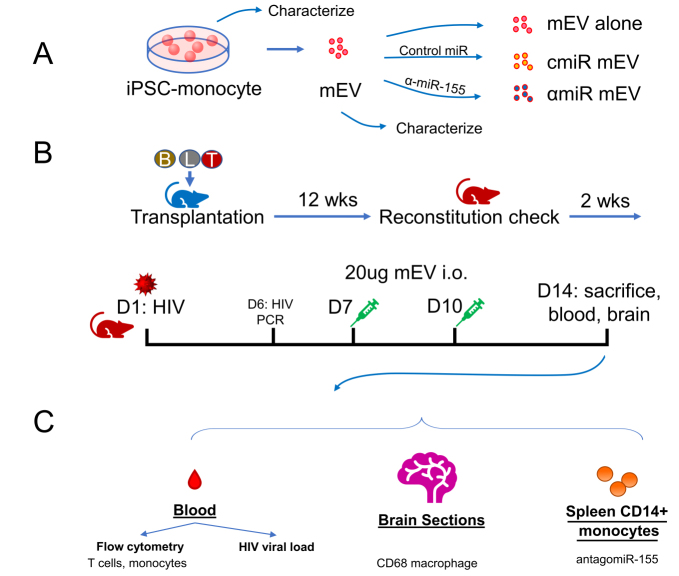
Experimental outline. (A) Preparation of iPSC-monocyte-derived extracellular vesicles (mEV), (B) preparation of HIV-infected Bone marrow Liver Thymic (BLT) mice, mEV treatment and (C) collection of samples. cmiR mEV: mEVs transfected with control miRNA; αmiR mEV: mEVs transfected with antagomiR-155; i.o.: intraocular injection; wks: weeks; D: day.

Human iPSC differentiated monocytes (ATCC-ACS-7030) were a gift from the American Type Culture Collection (ATCC, Manassas, VA, USA). Frozen iPSC-monocytes were thawed at 37 °C and washed and cultured in RPMI-1640 with 10% exosome-depleted fetal bovine serum (FBS, Thermo Fisher Scientific, Waltham, MA, USA). A low dose of 500 pg/mL rhMCSF (RandD Systems, Minneapolis, MN) was used to maintain monocyte viability. Cells were cultured at 37 °C with 5% CO_2_ for 48 hrs. Conditioned media were collected. EVs were purified with ExoQuick-TC (System Biosciences, SBI, Palo Alto, CA, USA) as described by the manufacturer [Figure 1A]. EVs were then stored at -80 °C until use. 

### Characterization of mEVs by NTA, electron microscopy and tetraspanins

Nanoparticle tracking analysis (NTA) was performed on the mEV samples to determine the size and particle number. Data were generated using a NanoSight LM10 instrument (Malvern Instruments, Malvern, United Kingdom) with a 405 nm laser-equipped sample chamber as previously described^[4]^. Results were analyzed using NTA 3.3 software. Each sample analysis consisted of three 40 s video recordings. Mode particle sizes were reported due to the skewed distributions.

mEVs were characterized by transmission electron microscopy (TEM). In brief, eluted mEVs were fixed in 4% buffered paraformaldehyde (PFA) and deposited onto Formvar carbon-coated electron microscopy nickel grids for 5 min. The excess fluid was blotted off with #1 filter paper, and the grids were stained with saturated uranyl acetate solution (Ted Pella, Inc., USA) for 5 s. Excess fluid was then blotted off again, and the grids dried overnight. Visualization of EVs was performed using a Technai 10 transmission electron microscope (Field Electron and Ion Co. USA).

The relative amount of tetraspanin proteins CD63, CD81 and CD9 on the surface of the mEVs were evaluated using a U-PLEX human tetraspanin kit (MSD, Meso Scale Diagnostics, Rockville, MD, USA) in duplicate according to the manufacturer’s instructions. Analyses were done using a QuickPlex SQ 120 instrument (MSD) and Discovery Workbench^®^ 4.0 software. 

### Antisense miR-155-Cy5 and transfection

Antisense miR-155-5p (αmiR) sequence (5’-/5Cy5/ AACCC CUAUC ACGAU UAGCA UUAA-3’) and Control Oligo-1 miR (cmiR) (SBI) were submitted to Integrated DNA Technologies (Redwood City, CA), synthesized and labelled with Cy5 at the 5’ end. EVs were transfected with antisense miR-155 or Control Oligo-1 miRs with Exofect (SBI) following the manufacturer’s instructions. Transfected EVs were purified with ExoQuick-TC (SBI) and stored at -80 °C until use [Figure 1A].

### Luciferase assay

The plasmid containing 3’ UTR of human E2F2 (NM_004091.3, HMIT094972-MT05) was purchased from GeneCopoeia (Rockville, MD, USA). HEK293 cells were cultured in a 96-well plate at 4 × 10^4^/well. Exchanged media every other day until 80% confluence. Then cells were transfected with 0.1ug HMIT094972-MT05 plasmid using Lipofectamine 3000 (Thermo). Media was exchanged after 24hr. Half of a microgram of cmiR or αmiR were added to the wells. Supernatants were collected after 24 h of incubation at 37 °C. Dual luminescence assays were performed on the cell culture supernatants using Secrete-Pair Dual Luminescence Assay Kit (GeneCopoeia) per manufacturer protocol. Plates were read using a SpectraMax M5 plate reader (Molecular Devices, San Jose, CA, USA). The ratio of secreted Gaussia luciferase and secreted alkaline phosphatasse was reported.

### HIV infection of BLT mice and mEV treatment 

Humanized BLT mice were constructed and housed at The University of California, Los Angeles (UCLA) Humanized Mouse Core as previously described^[9]^. Animal use was approved by the UCLA IACUC (Protocol ARC-2010-038). The model was validated with flow cytometry as described^[10]^. Briefly, human fetal CD34+ liver cells and thymus tissue were transplanted into irradiated non-obese diabetic (NOD) severe combined immunodeficient (SCID) gamma mice. Mice were monitored daily for signs of toxicity. Human immune cell engraftments were verified after 12 weeks [Figure 1B]. Thirty mice were divided into six groups: HIV-uninfected non-treated controls (HIV- NT, *n *= 4), HIV-uninfected antagomir-155 mEV (HIV- αmiR, *n *= 4), HIV-infected non-treated (HIV+ NT, *n *= 5), HIV-infected mEV alone (HIV+ mEV, *n *= 6), HIV-infected control miR mEV (HIV+ cmiR, *n *= 6) and HIV-infected antagomir155 mEV (HIV+ αmiR, *n *= 5). 

The mice were infected with 500 ng (approximately 6.25 × 10^4^ infectious units) of HIV (NFN-SX, R-5 tropic) intravenously. HIV viral RNA in the peripheral blood was checked 6 days post infection (p.i.). Twenty micrograms of mEVs were injected intraocularly on day 7 and day 10 p.i. Two weeks p.i., the mice were sacrificed, and the blood was processed to evaluate HIV viral RNA, inflammatory response and the effects of EVs on the mice were analyzed. Brains were collected for immunohistochemistry staining [Figure 1B and C].

### Human peripheral blood mononuclear cells isolation, mEV treatment and flow cytometry

To evaluate the propensity of mEVs to enter different cell types in the peripheral blood and particularly monocytes, we performed an mEV entrance experiment. Peripheral blood mononuclear cells (PBMCs) were enriched from a healthy donor as previously described^[11]^. In an ultra-low attachment 6-well plate (Corning, Kennebunk, ME, USA), 6 × 10^6^ PBMCs were cultured in RPMI-1640 supplemented with 10% FBS at 2 × 10^6^/mL. Ten µg of mEVs transfected with Cy-5 labelled antagomir-155 were added. Cells were incubated for 24 h and sampled at 2 h, 8 h and 24 h. Cells were immediately stained with CD8-FITC, CD4-PerCP-Cy5.5, CD14-PE, CD16-BV421 (all from BD, Becton, Dickinson and Company, Franklin Lakes, NJ, USA) for 30 min at room temperature (RT). Cells were then washed and fixed in 2% PFA for 10 min at RT, followed by washing. Flow cytometry analyses were performed on a FACSAria II flow cytometer (BD) and data were analyzed with FlowJo software (BD). At least 10,000 cells were collected for each sample. Gates were set using isotype antibodies (BD).

### HIV viral RNA

Blood from the HIV-infected mice was collected with EDTA anticoagulant from retro-orbital biweekly bleeding or heart puncture at sacrifice. Blood was spun at 1200 g to collect plasma supernatant. Cell-free plasma viral RNA was purified using a QIAamp Viral RNA Mini Kit (Qiagen, Germantown, MD, USA). HIV RNA was quantified by real-time reverse-transcription polymerase chain reaction (RT-PCR) using TaqMan RNA-To-Ct One-Step reagents (Thermo) with primers HIV_F: 5’-CAATG GCAGC AATTT CACCA-3’ and HIV_R: 5’-GAATG CCAAA TTCCT GCTTG A-3’ and a probe hybridizing to HIV1 NL4-3 HIV probe: 5’-[6FAM] CCCAC CAACA GGCGG CCTTA ACTG [Tamra-Q]-3’. 

### RT-PCR analysis for miRNA 

Five microliters of EV solutions were lysed in 350µL Qiazol (Qiagen) and total RNA was isolated using miRNeasy Mini kit (Qiagen). Two microliters of total RNA were reversely transcribed using a Taqman MicroRNA RT kit (Thermo). Taqman assay for human miR-155-5p (Thermo) was used for RT-PCR. Taqman advanced master mix was used for PCR. Assays were performed and analyzed on a ViiA7 instrument (Thermo) in triplicate. Relative expressions to RNU6 were reported.

### Antibodies and flow cytometry for mouse PBMCs 

The following antibodies were used in flow cytometry: CD45-V500 (clone HI30), CD3-BV786 (clone OKT3), CD4-Vioblue (clone RPA-T4), CD8-APC780 (clone SK1), CD19-APC, CD15-PerCP-Cy5.5, CD14-ECD, CD16-AF700, CD11b-PE-Cy7, CD209-FITC, CCR5-PC5, CD163-BV605, and major histocompatibility complex, class II, DR (HLA-DR)-PE (all BD). The cells were acquired using an LSRFortessa flow cytometer and FACSDiva software (BD). Data were analyzed using FlowJo software. At least 10,000 cells were acquired for each analysis, and each representative flow plot was repeated more than three times. 

### Spleen monocyte isolation

Human CD14+ monocytes were sorted from splenocytes through a human CD14 microbead kit (Miltenyi, Gaithersburg, MD, USA. Cat#130-050-201). We double-checked the CD14 clone (TÜK4) in this kit, and it does not have cross-reaction with mouse species.

### Immunostaining of brain sections

Mouse brains were collected and fixed in 2% paraformaldehyde. After 72 h, the tissues were washed with water and placed in 70% ethanol. Brains were embedded in paraffin and sectioned. Briefly, sections were deparaffinized and rehydrated. Antigen retrieval was performed by boiling for 30 min. Slides were treated with an endogenous peroxidase blocking solution (Vector, USA), followed by another block with 3% bovine serum albumin. Anti-CD68 rabbit monoclonal antibody (Abcam, USA, 1:500) was incubated at 4 C overnight in a humidified chamber. Anti-rabbit IgG secondary antibody was added (Vector), followed by diaminobenzidine substrate for 5 min and counterstained with hematoxylin. All slides were read blinded by a single reader.

### Statistical analysis

Data are reported as mean ± standard deviation unless otherwise specified. Wilcoxon signed-rank test was used to compare differences between two group means, analysis of variance (ANOVA) or Kruskal-Wallis rank sum test was used to compare differences between three or more group means where appropriate. Count data were compared with negative binomial regression. *P* < 0.05 was considered significant. All statistical analyses were performed with R (version 4.1.1).

### RESULTS

### iPSC-monocytes express CD14

**Figure 2:**
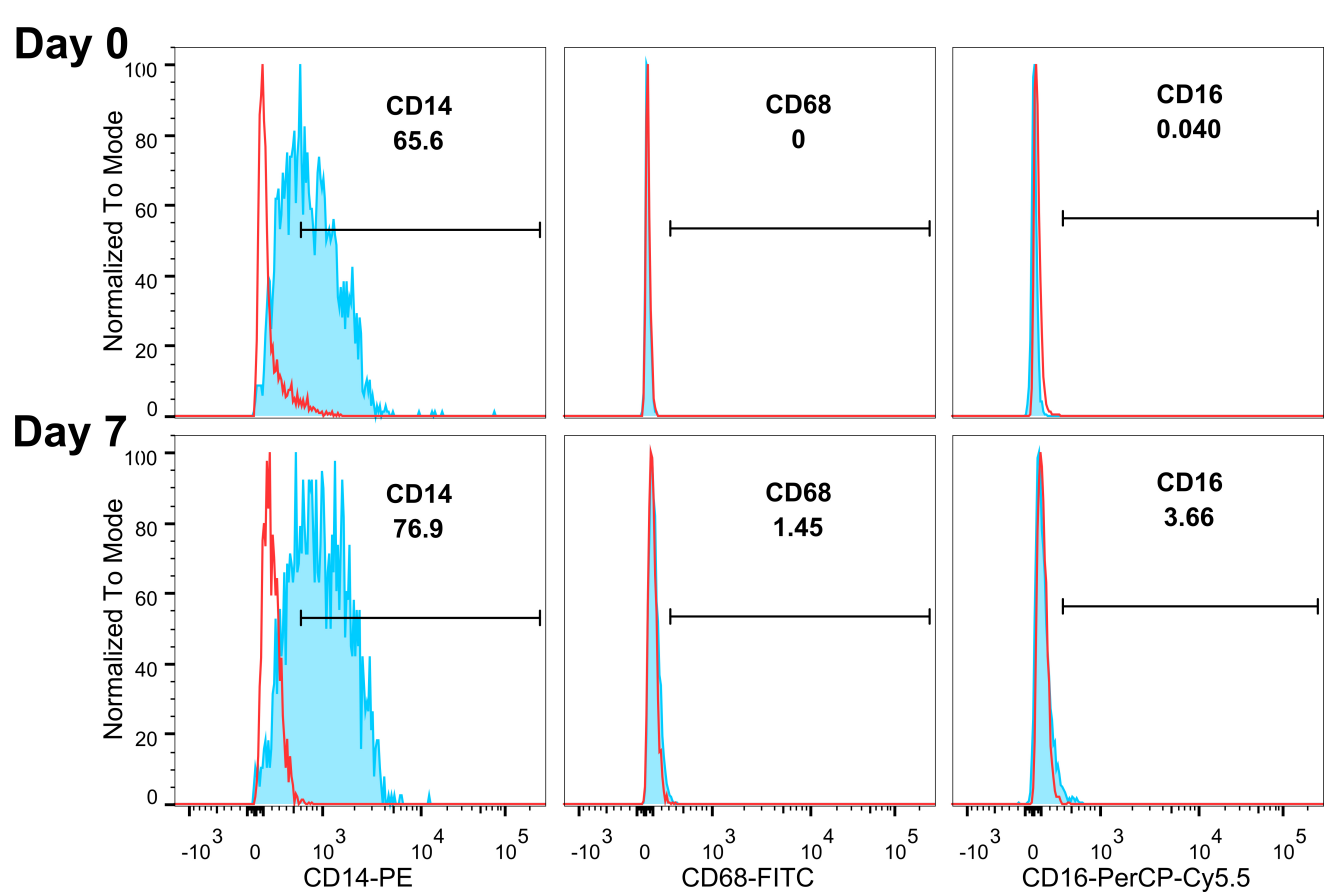
iPSC-monocytes remain CD14^+^ over time. Flow cytometry analysis show monocytes continue to express CD14 and not CD68 or CD16 on Days 0 and 7. Red lines denote isotype controls, blue lines with shaded areas denote corresponding antibody staining. Gating shows in percent of the parent population.

iPSC-derived monocytes expressed CD14 by flow cytometry from 65.5% on day 0 to 76.9% on day 7 [Figure 2]. CD68 and CD16 were negative on day 0 and slightly increased on day 7; the majority of the cells remained CD16 and CD68 negative.

### iPSC-monocyte EV characterization

**Figure 3:**
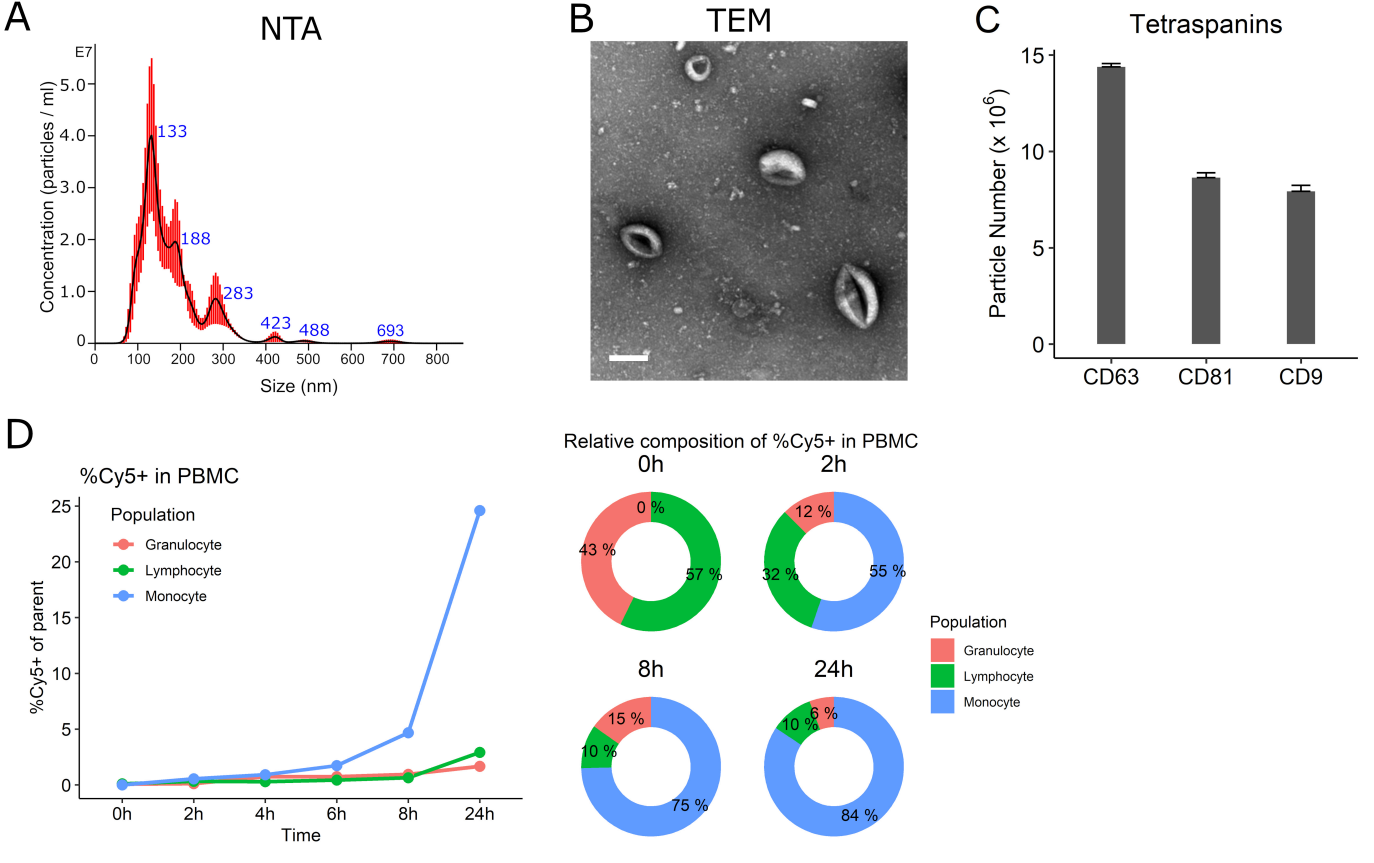
Characterization of iPSC-monocyte EVs (mEV). (A) Representative nanoparticle tracking analysis (NTA) showing the majority of mEVs approximately 130nm. (B) Transmission electron microscopy (TEM) of mEVs. Scale bar = 100 nm. (C) mEVs express tetraspanins CD9, CD63 and CD81. (D) Cy-5-labeled mEVs preferentially enter peripheral blood mononuclear cells (PBMC) monocytes over time.

NTA characterization showed the mEVs had an average mode size of 150.8 nm (standard error 19.6 nm) and a major peak at 133nm [Figure 3A]; the concentration was 1.27 ± 0.115 × 10^9^ particles/mL. The protein concentration was 200 µg/mL by absorbance at 280nm using a spectrophotometer measurement. Typical donut-shaped vesicles were visualized by TEM [Figure 3B]. mEVs expressed abundant CD63 (14.4 ± 0.17 × 10^6^ particles/mL) with CD81 (8.6 ± 0.25 × 10^6^ particles/mL) and CD9 (7.9 ± 0.31 × 10^6^ particles/mL) also present [Figure 3C]. When mEVs were incubated with PBMCs, mEVs entered lymphocytes in less than 2 h followed by monocytes preferentially over time. About 84% of the monocyte population were Cy5 positive after 24 h of treatment [Figure 3D].

### miRNA cargo and antagomir-155

**Figure 4:**
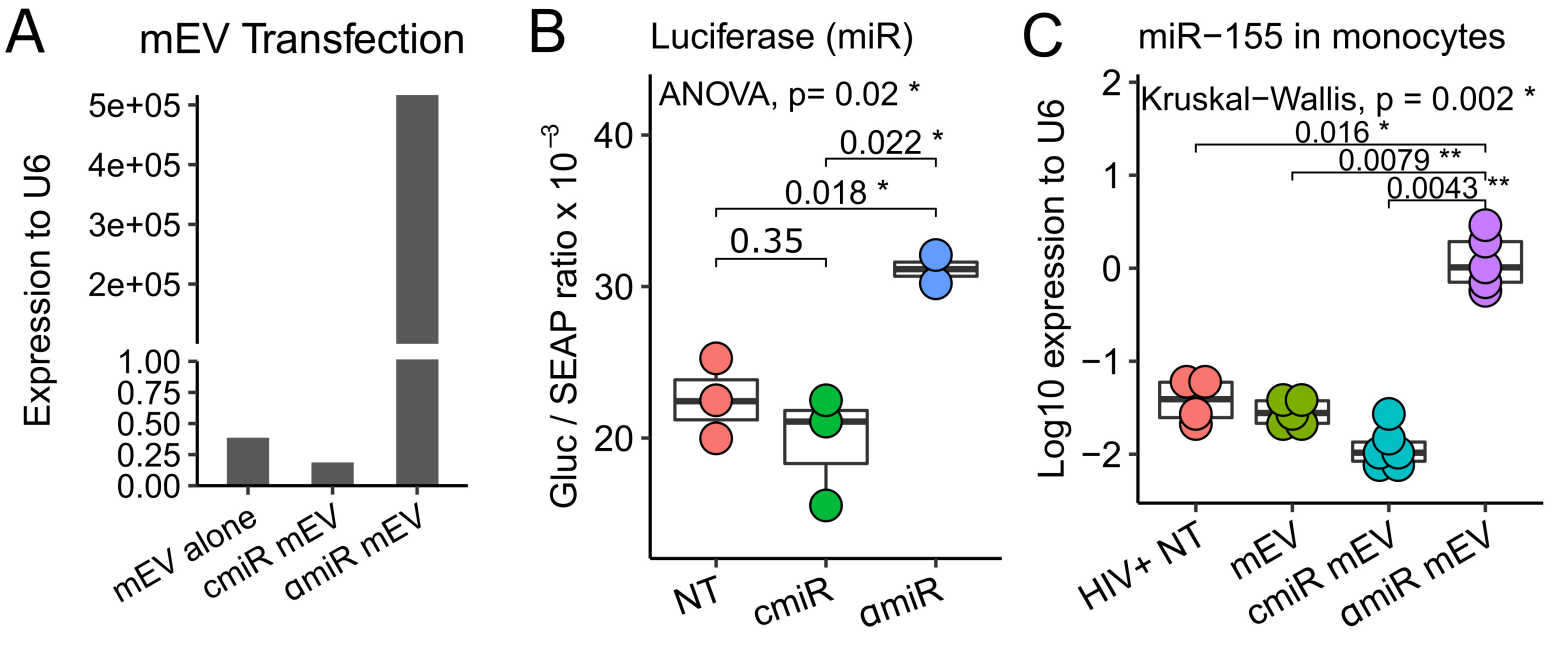
Antagomir-155 loading, targeting and delivery. (A) mEVs transfected with antagomiR-155 (αmiR mEV) showed expression of miR-155 compared to mEV alone and mEVs transfected with Control Oligo-1 miR (cmiR mEV). (B) αmiR, which was transfected into luciferase-plasmid-transfected HEK293 cells, successfully neutralized miR-155 and increased downstream luciferase expression. The ratio of *Gaussia* Luciferase (GLuc) and Secreted Alkaline Phosphatase (SEAP) is reported. (C) Mouse spleen CD14+ monocyte antagomiR-155 expression increases indicating successful delivery of αmiR mEV into monocytes. NT: No mEV treatment; αmiR: antagomiR-155; cmiR: control miRNA. *P < 0.05, **P < 0.01.

Because the antisense sequence of miR-155 is complementary to the sense sequence, both sense and anti-sense miR-155 were detected by the RT-PCR assay. αmiR mEVs showed very high levels of sense / anti-sense miR-155 expression (5.2 × 10^5^ fold relative to U6) compared to mEV alone (0.39 fold) and cmiR mEVs (0.19 fold) due to transfection of the antagomir-155 [Figure 4A]. The targeting of miR-155 by antagomir-155 was validated with a luciferase assay. A 58% increase in antagomir-155 treatment (31.1 ± 1.3 Gluc/SEAP ratio) compared to control miR treatment (19.7 ± 3.7 Gluc/SEAP ratio) showed that the constitutive inhibition effect from endogenous miR-155 to the downstream luciferase gene was partially reversed by antagomir-155 [Figure 4B]. To determine the entry of the αmiR mEVs into monocytes, we performed RT-PCR of miR-155 on CD14+ monocytes from the mouse spleen. The αmiR mEVs showed significant increase of miR-155 expression (1.427 ± 0.981 fold to RNU6) than HIV+ NT (0.0419 ± 0.0211, *P *= 0.016), mEV alone (0.029 ± 0.0087, *P *= 0.0079) or cmiR mEV (0.013 ± 0.0079, *P *= 0.0043) [Figure 4C].

### BLT mouse construction, HIV infection and mEV treatment

**Figure 5:**
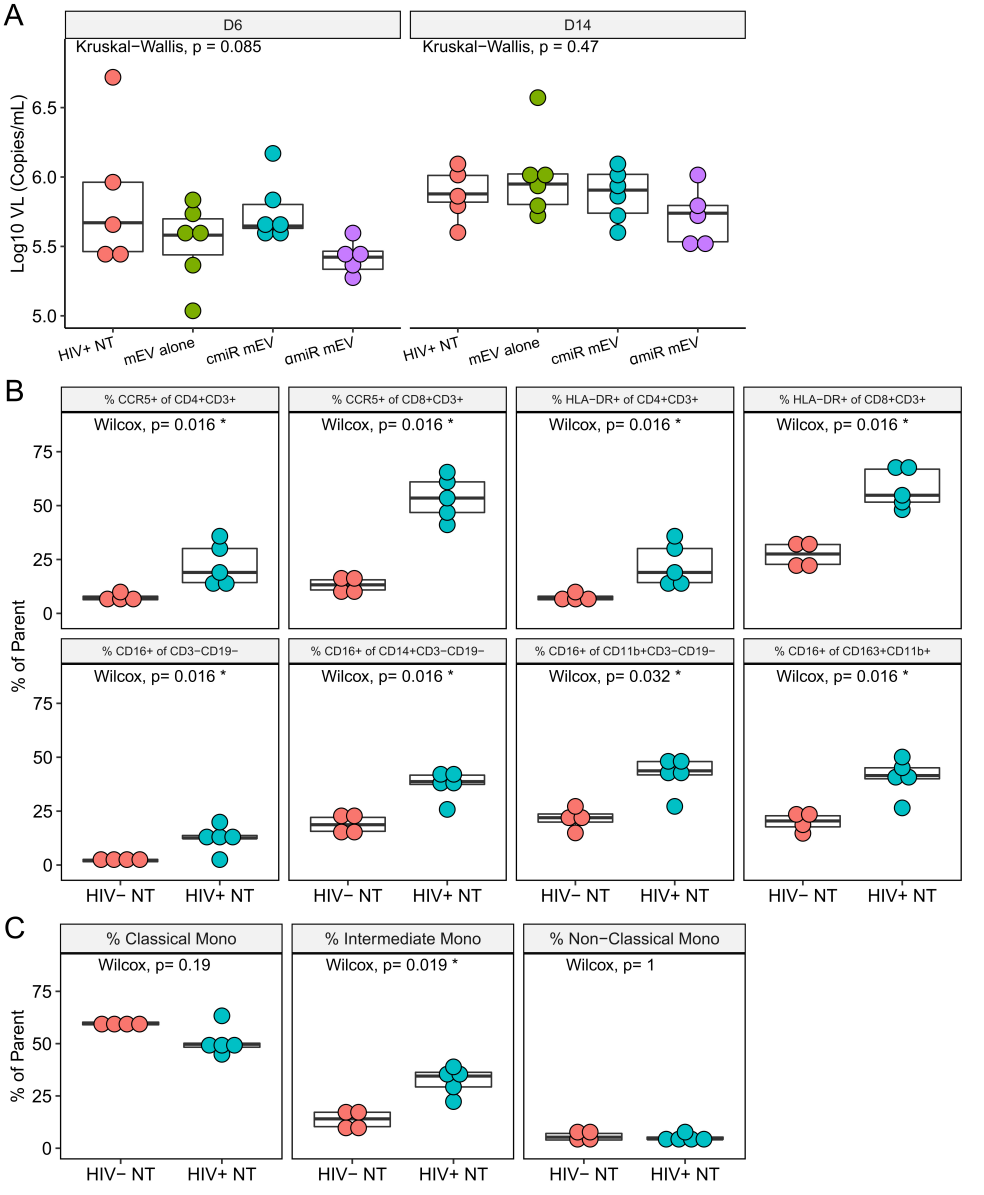
HIV-infected BLT mice. (A) Box plots for mouse plasma viral load on Days 6 and 14 post infection showed no difference among groups. (B) Frequencies of CCR5, HLA-DR and CD16 on PBMCs after HIV infection on Day 14. Parent populations are indicated in the titles of the panels. (C) Frequency of monocyte subsets in CD14+ monocytes. Parent population is CD14+CD3-CD19-.

After BLT mice were constructed as described previously^[9]^, reconstitution of human blood cells was confirmed by flow cytometry after 12 weeks following transplantation [Figure 1B], and all mice showed proper reconstitution of human PBMCs [Supplementary Figure 1]. One mouse in the HIV+ αmiR group died during HIV infection and was eliminated from the group. All other mice tolerated two doses of mEV injection with no side effects. HIV viral load in the peripheral blood was measured on day 6 p.i. and after sacrifice on day 14. There was no significant difference among groups on either day 6 (*P *= 0.085) or day 14 (*P *= 0.47) [Figure 5A]. Fourteen days after HIV infection, %CCR5+ increased in CD4 T cells (7.4 ± 1.7 for HIV- *vs. *22.5 ± 9.9 for HIV+, *P* = 0.016) and CD8 T cells (13.2 ± 3.9 for HIV- *vs. *53.6 ± 10.0 for HIV+, *P* = 0.016). %HLA-DR+ significantly increased in CD4 T cells (7.4 ± 1.7 for HIV- *vs. *22.5 ± 9.9 for HIV+, *P* = 0.016) and CD8 T cells (27.1 ± 5.8 for HIV-* vs. *58.0 ± 9.2 for HIV+, *P* = 0.016). The increases of CCR5 and HLA-DR on CD4+ or CD8+ T cells are consistent with previous reports^[9]^, confirming that HIV successfully activated human PBMCs in the BLT mouse model. The %CD16+ cells increased in various cell populations indicating activation of M/Mφ, which includes the non-B non-T cells (CD3-CD19-) (2.1 ± 0.7 for HIV- *vs. *12.4 ± 5.8 for HIV+, *P* = 0.016), CD14+ monocytes (19.1 ± 4.5 for HIV- *vs. *37.2 ± 6.7 for HIV+, *P* = 0.016), CD11b+ macrophages (21.7 ± 5.3 for HIV- *vs. *41.7 ± 8.9 for HIV+, *P* = 0.032) and CD163+CD11b+ activated macrophages (20.1 ± 4.4 for HIV- *vs. *40.6 ± 8.8 for HIV+, *P* = 0.016) [Figure 5B]. Frequency of intermediate monocytes (13.5 ± 4.4 for HIV- *vs. *32.3 ± 6.6 for HIV+, *P* = 0.019) increased while classical (*P *= 0.19) and non-classical (*P *= 1) monocytes did not change [Figure 5C]. Median fluorescent intensity (MFI) analyses of CCR5, HLA-DR and CD16 expression on the CD4 or CD8 T cell populations showed similar results [Supplementary Figure 2].

### mEV altered surface marker expressions in monocytes and T cells with or without antagomir-155

**Figure 6:**
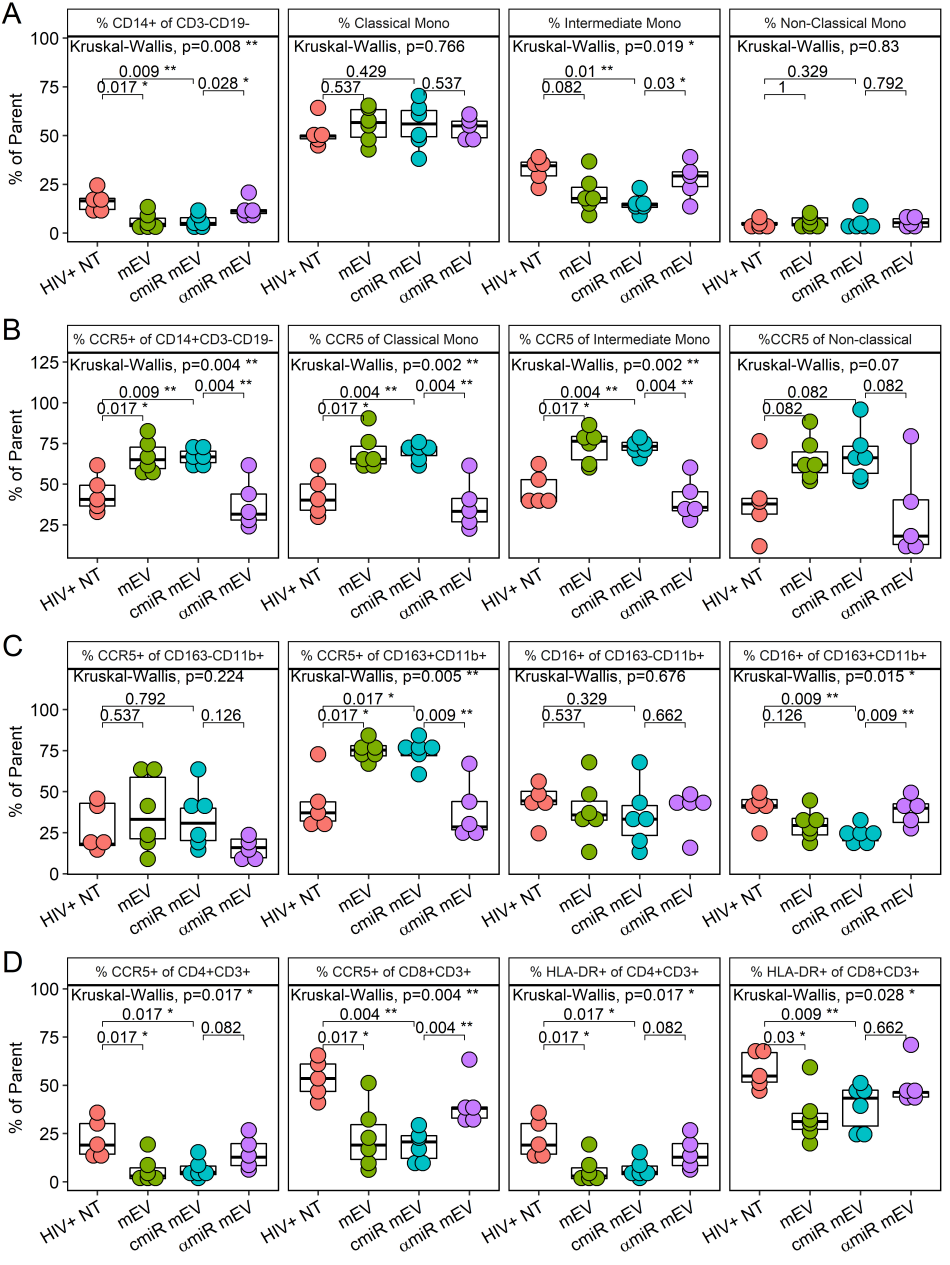
PBMC profile of iPSC-monocyte EV (mEV) treated HIV+ BLT mice. (A) %CD14+ of CD3-CD19-, classical monocytes (CD16-CD14++), intermediate monocytes (CD16+CD14++) and non-classical monocytes (CD16++CD14+) (B) %CCR5 expression of overall monocytes (CD14+CD3-CD19-), classical monocytes (CD16-CD14++), intermediate monocytes (CD16+CD14++) and non-classical monocytes (CD16++CD14+) (C) %CCR5 and %CD16 of macrophages including M1 macrophages (CD163-CD11b+CD3-CD19-) and M2 macrophages (CD163+CD11b+CD3-CD19-) (D) %CCR5 and %HLA-DR of CD4+ or CD8+ T cells.

The CD14 population decreased after mEV treatment in PBMCs (16.4 ± 5.3 for HIV+NT *vs.* 6.1 ± 4.1 for HIV+ mEV alone, *P* = 0.017) and partially normalized with antagomir-155 (6.0 ± 3.3 for cmiR mEV and 12.7 ± 4.5 for αmiR mEV, *P* = 0.028) [Figure 6A]. Intermediate monocytes trended lower (32.3 ± 6.6 for HIV+NT *vs.* 20.3 ± 9.5 for HIV+ mEV alone, *P* = 0.082) and were partially normalized by antagomir-155 (14.7 ± 4.4 for cmiR mEV and 27.6 ± 9.1 for αmiR mEV, *P* = 0.03). Alternatively, classical (*P *= 0.77) and non-classical (*P *= 0.83) monocytes did not show significant changes with mEV treatments [Figure 6A].

mEVs alone activated CCR5 on CD14+ monocytes (44.5 ± 11.2 for HIV+NT *vs. *67.0 ± 10.0 for HIV+ mEV alone, *P* = 0.017), classical CD14+ CD16- monocytes (43.2 ± 13.0 for HIV+NT *vs. *69.8 ± 11.5 for HIV+ mEV alone, *P* = 0.017), intermediate CD14++CD16+ monocytes (46.9 ± 10.6 for HIV+NT *vs. *73.2 ± 10.7 for HIV+ mEV alone, *P* = 0.017) and was trending on non-classical CD14+CD16++ monocytes (39.9 ± 23.26 for HIV+NT *vs. *65.5 ± 13.0 for HIV+ mEV alone, *P* = 0.082) [Figure 6B]. The treatment with antagomir-155 normalized the increase on CD14+ monocytes (67.0 ± 5.2 for cmiR mEV and 37.6 ± 14.8 for αmiR mEV, *P* = 0.0043), on classical monocytes (70.4 ± 5.0 for cmiR mEV and 37.1 ± 15.4 for αmiR mEV, *P* = 0.043), on intermediate monocytes (72.8 ± 4.3 for cmiR mEV and 40.9 ± 13.1 for αmiR mEV, *P* = 0.0043) and was trending on non-classical monocytes (68.0 ± 16.4 for cmiR mEV and 32.3 ± 28.8 for αmiR mEV, *P* = 0.082) [Figure 6B]. MFI analyses of CCR5 expression on these monocyte populations showed similar results [Supplementary Figure 3A].

The %CCR5+ increased on CD163+CD11b+ M2-type macrophages (42.6 ± 16.8 for HIV+NT *vs. *75.1 ± 6.6 for HIV+ mEV alone, *P* = 0.017) with mEV treatment and decreased with antagomir-155 mEV (74.0 ± 7.8 for cmiR mEV and 38.1 ± 18.6 for αmiR mEV, *P* = 0.0087) in HIV-infected mice [Figure 6C]. CCR5 had no significant changes on CD163-CD11b+ M1 macrophages. CD16 did not show a significant decrease with mEV treatments alone (*P* = 0.13), but showed a decrease with cmiR mEV (*P *= 0.0087) and a partial normalization on M2 macrophages with antagomir-155 (23.3 ± 5.0 for cmiR mEV and 38.1 ± 8.3 for αmiR mEV, *P* = 0.0087) [Figure 6C]. HLA-DR expression on M/Mφ did not show significant differences between groups (data not shown). MFI analyses of CCR5 and CD16 expression on the macrophage populations showed similar results except CD16 was trending low by mEV alone compared to HIV+ NT and trending increase by antagomir-155 compared to control miR [Supplementary Figure 3B].

mEVs alone also suppressed CCR5 expression in CD4+ (22.5 ± 9.9 for HIV+NT *vs.* 6.0 ± 6.8 for HIV+ mEV alone, *P* = 0.017) and CD8+ T cells (53.6 ± 10.0 for HIV+NT *vs. *22.9 ± 16.6 for HIV+ mEV alone, *P* = 0.017) and HLA-DR expression on CD4 (22.5 ± 9.9 for HIV+NT *vs. *6.0 ± 6.8 for HIV+ mEV alone, *P* = 0.017) and CD8+ T cells (58.0 ± 9.2 for HIV+NT *vs.* 34.0 ± 13.6 for HIV+ mEV alone, *P* = 0.03). The decrease of CCR5 on CD8+ T cells with mEVs alone in HIV+ NT mice was normalized with αmiR mEV treatment (19.0 ± 8.1 for cmiR mEV and 40.9 ± 12.9 for αmiR mEV, *P *= 0.0043) [Figure 6D]. Trending normalizations were also observed with CCR5 (*P *= 0.082) and HLA-DR (*P *= 0.082) on CD4+ T cells but not HLA-DR on CD8 T cells (*P *= 0.66). MFI analyses showed mEV alone suppressed CCR5 on CD8 T cells and trending on CD4 T cells, while HLA-DR was trending low on both CD4 and CD8 T cells. Antagomir-155 normalized CCR5 MFI on both CD4 and CD8 T cells while HLA-DR did not [Supplementary Figure 3C].

### CD68 immunohistochemistry staining

**Figure 7:**
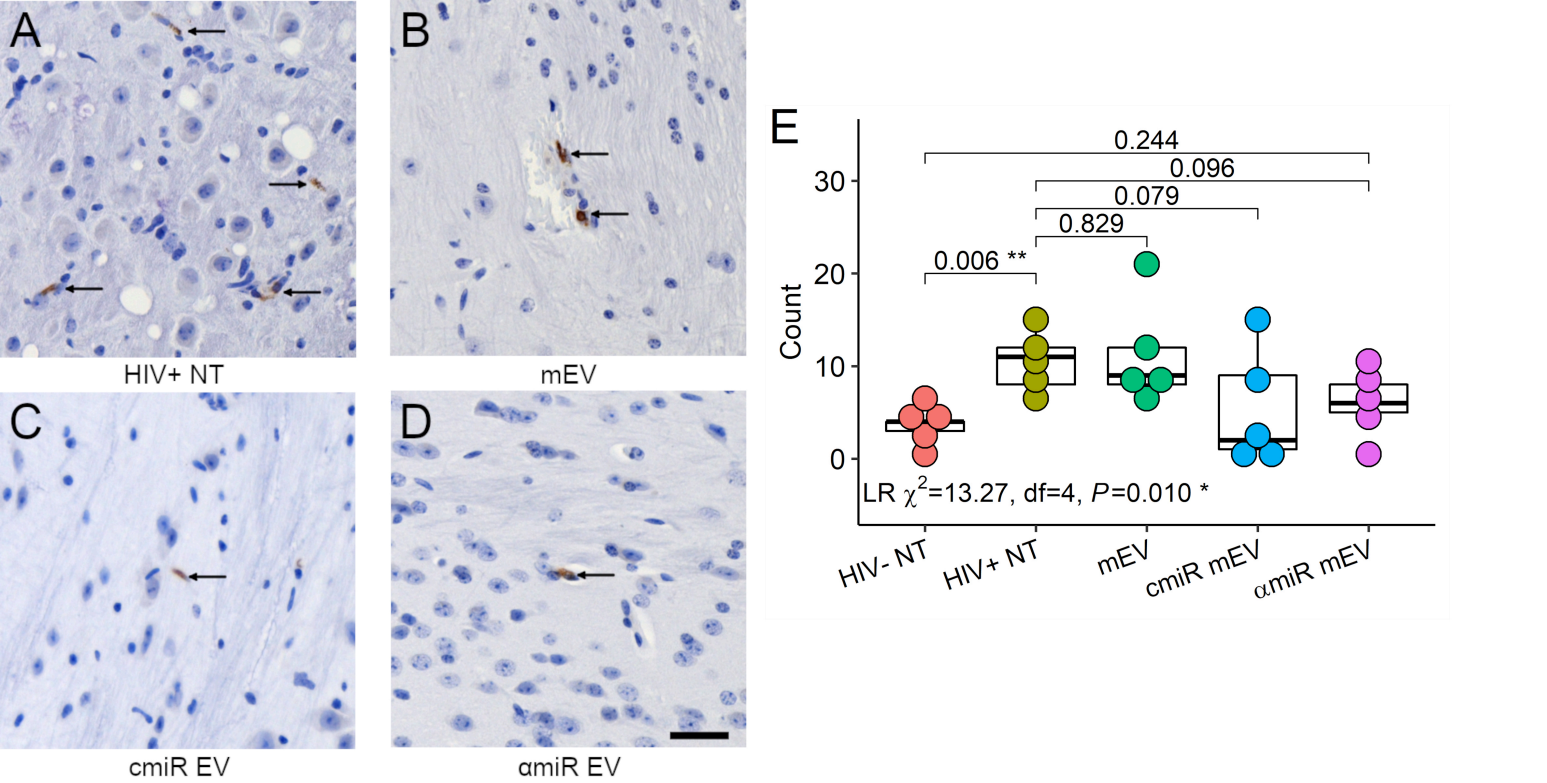
CD68 immunohistochemistry staining of the mouse brains. (A) HIV+ non-treated, (B) HIV+ and treated with mEV alone, (C) HIV+ and treated with cmiR mEVs, (D) HIV+ and treated with αmiR mEVs, arrows indicate positive CD68 staining. 20X. Scale bar = 25 µm. (E) CD68 positive cell counts. Group-wise counts were compared with negative binomial regression. LR: likelihood ratio test for negative binomial regression. *P < 0.05, **P < 0.01.

Mouse brain sections were stained for CD68 proteins [Figure 7A-D]. CD68 positive cell counts showed significant differences using negative binomial regression analysis (likelihood ratio *Χ*^2^ = 13.27, df = 4, *P *= 0.01). CD68+ cell counts increased in HIV-infected mouse brains compared to HIV-uninfected mice (3.6 ± 1.8 for HIV- NT, 10.6 ± 3.2 for HIV+ NT, *P *= 0.006) and no significant changes with mEV, cmiR or αmiR mEV treatments [Figure 7E].

### DISCUSSION 

Pluripotent stem cell-derived monocytes provide a uniform cell source with the ability to scale up as well as minimize human donor variations. Extracellular vesicles generated by these stem cell monocytes can be used as a tool for transferring cargo to other cells and targeting monocytes. Using EVs instead of cells to carry agents avoids drawbacks of stem cell treatment such as malignant transformation^[12,13]^. iPSC-derived monocytes showed monocytic morphology and cell surface markers such as high CD14, with low CD16 and CD68. This phenotype could be maintained for at least 7 days in culture, which makes it a good source of EVs*. *We chose stem cell-derived monocytes to increase the chance of EVs entering and delivering cargo to peripheral monocytes. 

EVs produced directly from stem cells have been used as therapeutic tools in different studies. Reports show that stem cell-derived EVs alleviated colitis^[14]^, attenuated aging-associated vascular endothelial dysfunction^[15]^, prevented allergic airway inflammation^[16]^ and promoted repair of cardiac infarction^[17]^ in mouse models. There are also various reports on engineered-parent cell generated miRNA-loaded EVs. EVs derived from stem cells transfected with miRNA mimics or antagomirs ameliorated spinal cord injury^[18]^ and microglial activation from brain injury^[19]^, promoted selective regeneration in ischemic hearts^[20]^ or alleviated systemic sclerosis^[21]^ in mouse models. We chose to transfect EVs directly due to the short lifespan of iPSC-monocytes and ease of manipulation.

In our previous studies on monocyte activation and the subsequent effect on their EVs, we found that monocytes treated with interferon/LPS released EVs that transferred functional miRs to human endothelial cells. miR-155, miR-146a, miR-146b and miR-125a-5p were significantly increased and miR-222 was significantly decreased^[5]^. We further determined that this activation was through the toll-like receptor 4/MYD88 innate immune signal transduction adaptor (MYD88) pathway that activates nuclear factor-κB^[5]^. We chose to transfect the antagomir-155 into iPSC monocyte-derived EVs to determine the effect on HIV-infected humanized mice with the hypothesis that the monocyte EVs with antagomir-155 would decrease neuroinflammation, possibly viral load and peripheral activation. miR-155 is well characterized, rapidly released in response to infections and injury, and is associated with pathological processes including inflammation and immune responses (Reviewed Ref.^[6,22]^). miR-155 can push myeloid cells to a pro-inflammatory phenotype^[23]^ that can culminate in neuroinflammation. Knockout of miR-155 reduced macrophage-mediated neuron dysfunction^[24]^ and death in mice^[25]^. The mEVs alone or with a scrambled control miR decreased T cell and increased monocyte activation. These divergent effects may be due to the constitutive expression of miR-155 in the EVs and its competing effects on these cell types. When an antagomiR-155 EV was introduced, there was a setback to HIV infection without treatment. However, there was a trend toward a decrease in the %CCR5+ on the non-classical monocytes that translated to a trend in decreased macrophage brain infiltration. These results may suggest an miR redirecting of migration^[26]^, in this case, from the brain to other tissues. While the exosomes may have delivered the miR-155 antagomir to monocytes, there was no significant decrease in activation markers. 

In this HIV-infected BLT humanized mouse model, we observed no differences in viral load before or after EV treatment suggesting that these mEVs do not act against HIV replication directly within this time period. CCR5 is a major HIV coreceptor expressed on T cells and monocytes. CCR5 also plays an important role in T cell and M/Mφ migration including infiltration of the central nervous system (CNS). Increased CCR5 in HIV infection is associated with amyloidosis, tau pathology, neurodegeneration, and blood-brain barrier alterations^[27]^. We observed an increase of CCR5 on monocytes after HIV infection but not on T cells. miR-155 expression in T cells regulates CCR5 and C-X-C motif chemokine receptor 4 and is central to T cell migration into organs^[28]^. In multiple sclerosis lesions, T cells that overexpress CCR5 migrate into lesions and the non-migratory T cells express low levels of CCR5^[29]^. The discrepancy between monocytes and T cells may be due to the difference in the timing of migration between monocytes and T cells. Intermediate monocytes increase and transmigrate across the blood-brain barrier after HIV infection^[30]^. CCR5 is associated with the CD16+ intermediate monocytes in HIV-infected individuals and a CCR5 antagonist improved neurocognitive performance in a small cohort, suggesting suppression of monocyte migration^[31]^. The CCR5 antagonist also blocked T cell chemotaxis *in vitro*. CD14+CD16+ monocytes are the subset of intermediate and non-classical cells that have a pro-inflammatory phenotype and migrate into tissues and the brain^[32,33]^. In this BLT model, the mEVs alone decreased the percent of intermediate monocytes, suggesting that treatment with mEVs had an effect on myeloid differentiation^[34]^.

Significantly, this pilot study showed that EVs can be loaded with cargos, transported to target cells and have biological effects *in vivo* including alteration of T cell and monocyte activation markers with a trend toward decreasing M/Mφ infiltration to the brain. EVs have the advantage of crossing biological barriers, delivering anti-inflammatory cargo to recipient cells^[35]^, thereby influencing peripheral and neuroinflammation. This would have implications for other neuroinflammatory pathologies. Alternatively, there are several limitations in this study including a small number of animals and short acute HIV infection period. mEVs were in the mice for only 7 intermediate days and this may have been an insufficient time or dose to have further effects. We used an intraorbital delivery of mEVs, and an intravenous route may have more robust effects. Future studies would require a closer examination of the timing of administration of the mEVs following infection and the optimization of the dosing (with and without antiretroviral therapy) to more closely assess the role of myeloid/T cell activation and antagomir-155 on HIV. 

Our results raise a number of interesting issues. Control mEV treatments decreased the number of CD16+ or intermediate monocytes in the periphery. These monocytes are thought to preferentially transmigrate to the brain in HIV infection^[33]^. These results suggest that targeting these monocytes would seem more beneficial than targeting the CCR5+ monocytes. The mEV treatments also caused a significant decrease in peripheral T cell activation. Our results show a differential expression of CCR5 and CD16 on monocytes, with mEVs decreasing the percentages of CD16+ monocytes and increasing the CCR5 expression on monocytes. Since CD14++/CD16+ monocytes preferentially transmigrate and seed the brain as a reservoir, targeting this subset in the periphery would be the best approach to limit CNS seeding^[33]^. The mEV treatments also caused an increase in %CCR5 of CD163+CD11b+ cells and a decrease in %CD16 of CD163+CD11b cells, again suggesting a divergent beneficial effect on monocytes. We posit that the mEV treatments decreased CD16+ monocytes and increased CCR5+ monocytes that may translate to higher migration to other tissues than the brain. These studies do not show an advantage in using antagomir-155 to reduce inflammation below HIV infection alone, but the numbers are small and the outliers may obscure significance. The contents of the mEVs need to be further investigated to determine these positive effects. To our knowledge, this is the first time an EV has been engineered to suppress an inflammatory miRNA and delivered into a humanized HIV mouse to modulate neuroinflammation. In conclusion, there appears to be value in treatment with monocyte EVs in reducing CD16+ monocytes, and although CCR5 was increased on monocytes in mEV-treated mice, this did not translate to an increase in migration to the brain.

## DECLARATIONS

### Copyright

© The Author(s) 2022.
