# Supplementary material for: Engineered induced-pluripotent stem cell derived monocyte extracellular vesicles alter inflammation in HIV humanized mice
Source: Extracell Vesicles Circ Nucl Acids. 2022 Apr 24;3(2):118–32. doi: 10.20517/evcna.2022.11 (PMC10104589; doi:10.20517/evcna.2022.11)
Supplement: Supplementary file 1 [file evcna-3-2-118-SupplementaryMaterials.pdf]

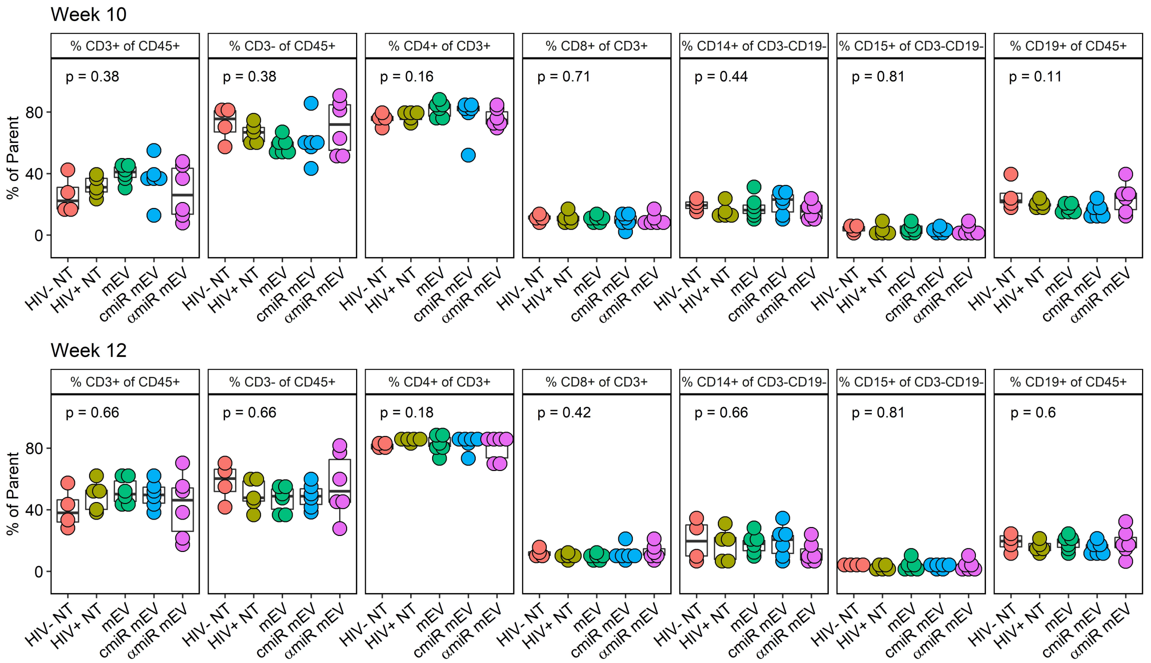

Supplementary Figure 1. Characterization of BLT mouse after 10 or 12 weeks of implantation by flow cytometry analysis on PBMCs showing that the animals were reconstituted with human T, B and myeloid cells. HIV- NT: HIV non-infected without mEV treatment; HIV+ NT: HIV infected without mEV treatment; cmiR mEV: HIV+ and treated with mEVs transfected with control miRNA; αmiR mEV: HIV+ and treated with mEVs transfected with antagomiR-155. PBMC: peripheral blood mononuclear cells.

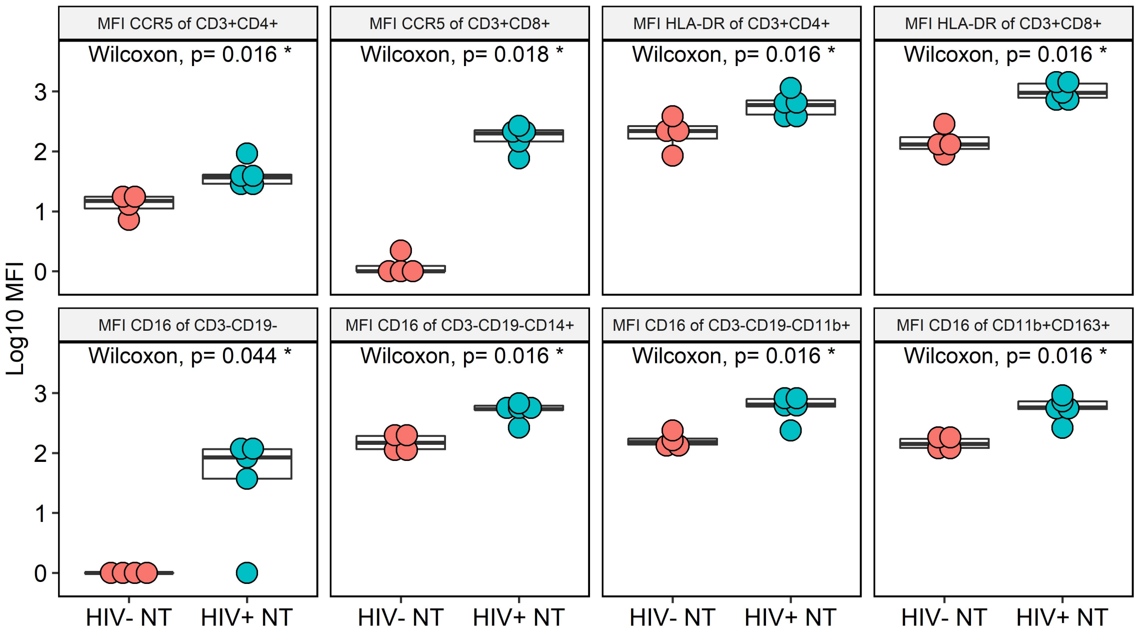

Supplementary Figure 2. Median fluorescence intensity (MFI) of CCR5, HLA-DR and CD16 increased showing PBMC activation after HIV infection at Day 14. NT: No mEV treatment; PBMC: peripheral blood mononuclear cells; CCR5: C-C chemokine receptor type 5; HLA-DR: Major Histocompatibility Complex, Class II, DR. * $P < 0.05$

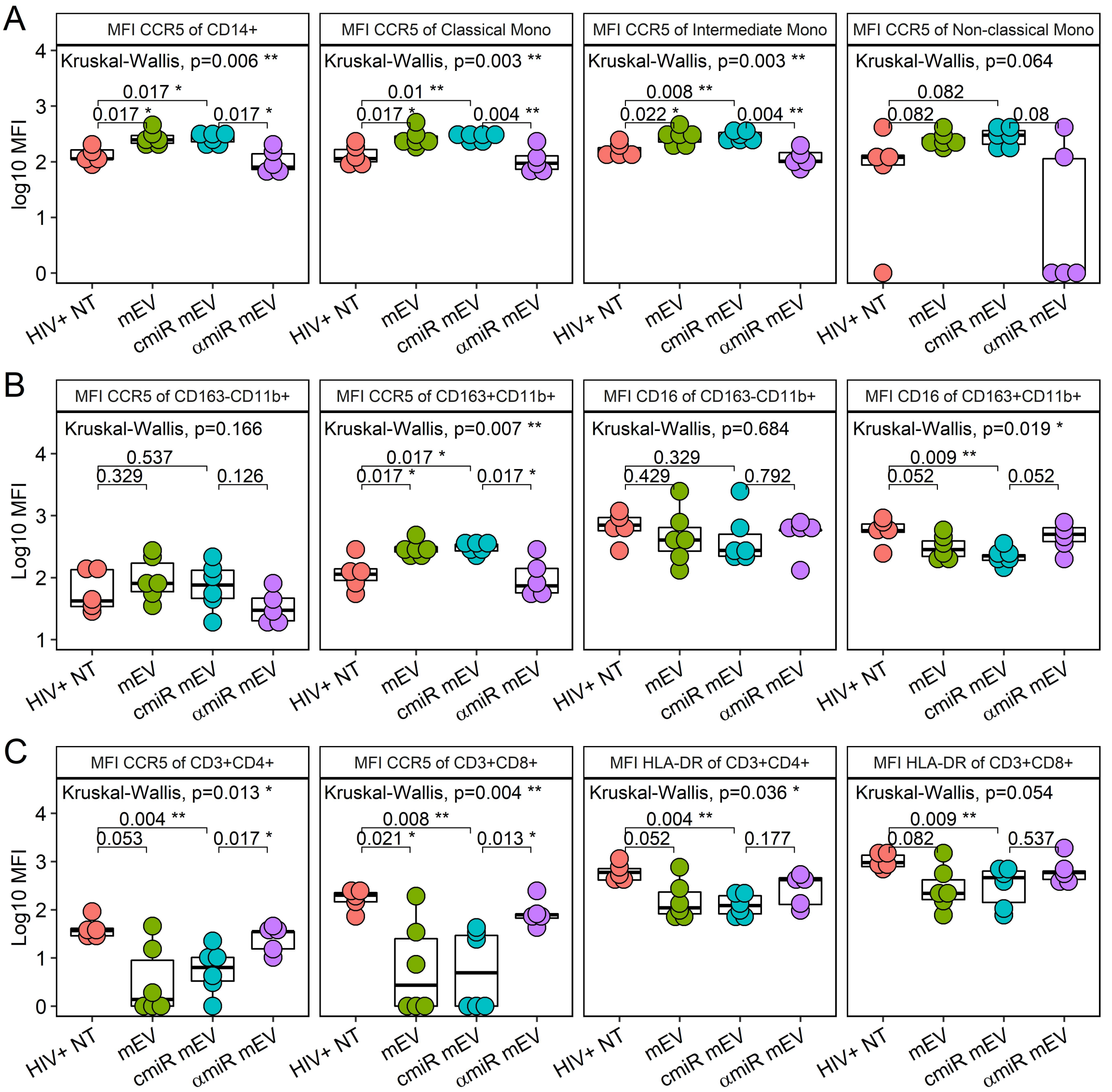

Supplementary Figure 3. PBMC profile of iPSC-monocyte EV (mEV) treated HIV+ BLT mice. (A) CCR5+ median fluorescent intensity (MFI) of overall monocytes (CD14+CD3-CD19-), classical monocytes (CD16-CD14++), intermediate monocytes (CD16+CD14++) and non-classical monocytes (CD16++CD14+) (B) MFI of CCR5+ and CD16+ of macrophages including M1 macrophages (CD163-CD11b+CD3-CD19-) and M2 macrophages (CD163+CD11b+CD3-CD19-) (C) MFI of CCR5+ and HLA-DR+ of CD4+ or CD8+ T cells. HIV+ NT: HIV+ without mEV treatment; cmiR mEV: HIV+ and treated with mEVs transfected with control miRNA; αmiR mEV: HIV+ and treated with mEVs transfected with antagomiR-155. PBMC: Peripheral blood mononuclear cells;

BLT: Bone Marrow Liver Thymic; CCR5: C-C chemokine receptor type 5; HLA-DR Major Histocompatibility Complex: Class II, DR. * $P < 0.05$, ** $P < 0.01$.
